# Correlation Between Obesity and Mental Health Status in Riyadh, Saudi Arabia

**DOI:** 10.7759/cureus.53976

**Published:** 2024-02-10

**Authors:** Khalid A Al Nasser, Yousaf K Alsaffar, Akram N Al Hazmi

**Affiliations:** 1 Family Medicine Department, King Saud Medical City, Riyadh, SAU

**Keywords:** depression, anxiety, bmi, mental health, obesity, correlation

## Abstract

Introduction: Recent studies show an increase in the incidence and prevalence of obesity worldwide. In Saudi Arabia, the prevalence of obesity, according to the latest studies, was estimated to be 24.7%. Rising rates of obesity are becoming a serious public health concern with well-documented physical and mental health consequences. Our study aims to measure the effect and the relationship between obesity and mental health status and to assess the impact on the quality of life in Riyadh, Saudi Arabia.

Methods: A cross-sectional observational study was conducted in Riyadh, Saudi Arabia. Data collection involved an electronic questionnaire encompassing patient demographics, their perspectives on obesity and its impact on mental health, as well as screening for common mental health disorders using GAD-2 and PHQ-9. Subsequently, the data were coded, entered, and analyzed utilizing both descriptive and inferential statistical methods, with the assistance of IBM Corp. Released 2015. IBM SPSS Statistics for Windows, Version 23.0. Armonk, NY: IBM Corp.

Results: A total of 480 adult Saudi participants were included in the current study. Most of them were males (61.5%) in the age group of 18 to 40 years (77.3%). 13.1% of the participants had a first-degree family history of psychiatric illness, and 10.6% had a previous medical history of psychiatric illness. 86% of the participants think that obesity has a negative effect on the quality of life and mental health, and 98.1% believe that losing weight and treating obesity will play a role in improving the quality of life and mental health in general. Regarding the prevalence of mental health issues according to the PHQ-2 score, 151 (31.5%) of the participants were positive, out of whom 47 (31.1%) had mild depression, and 147 (30.5%) of the participants were positive using the GAD-2, out of whom 41 (27.9%) had mild anxiety, and the same percentage had moderate anxiety. There was a significant association between BMI and a previous history of psychiatric illness among females (P = 0.044).

Conclusion: Obesity and a higher BMI were found to be associated with a higher prevalence of depression and anxiety among the study participants. About one-third of the total participants had depression, and another one-third had an anxiety disorder.

## Introduction

Obesity is a condition in which excess body fat accumulates to such an extent that it could negatively contribute to health [1]. It is recognized as a risk factor for various non-communicable diseases such as diabetes mellitus, hypertension, cardiovascular diseases, and musculoskeletal disorders, leading to a significant decrease in life quality and expectancy [2]. According to the World Health Organization (WHO), obesity is defined as a body mass index (BMI) greater than or equal to 30 kg/m^2^ and is classified into three different classes. BMI is a statistical index that uses a person's weight and height to estimate body fat in males and females of any age, calculated as BMI = weight (in kg)/height^2^ (in m^2^) [3].

Recent studies indicate a global increase in the incidence and prevalence of obesity [4]. In Saudi Arabia, the prevalence of obesity, according to the latest studies, was estimated to be 24.7% [5]. The escalating rates of obesity pose a serious public health concern with well-documented physical and mental health consequences [6]. Mental health is a state of well-being in which an individual realizes their own abilities, can cope with the normal stresses of life, can work productively, and can contribute to their community. Mental health, rather than just the absence of mental illness, encompasses the presence of positive characteristics [7]. In the realm of mental health illnesses, our primary focus is on depression and anxiety. Depression, a common mental disorder, affects an estimated 5% of adults globally and is characterized by persistent sadness, a lack of interest or pleasure in previously rewarding activities, disturbed sleep, and appetite changes [8]. Anxiety disorders involve excessive fear, worry, and related behavioral disturbances, causing significant distress or impairment in functioning [9]. A literature search retrieved 11 reviews on obesity and psychological factors, indicating a link between obesity and depression disorders [10]. A recent study in Al Kharj, Saudi Arabia, showed a significant association between depression and obesity among participants [11].

Our study aims to assess the effect of obesity on mental health status, its relation to depression and anxiety disorders, and its impact on quality of life. Additionally, we aim to evaluate the perception of Vision 2030 on the population's general health, considering protective measures, new facilities, sports projects, and events encouraging a healthy lifestyle.

## Materials and methods

A cross-sectional study was conducted in Riyadh, Saudi Arabia, utilizing a translated questionnaire distributed electronically for self-administration to enhance accessibility. This design was selected for its convenience and effectiveness in studying the research hypotheses and objectives. The survey was distributed in clinical-based settings across the primary healthcare facilities of the three health clusters, comprising two main sections.

The first section collected demographic data, including age, gender, nationality, education, BMI, a history of psychiatric disorders, and the use of antipsychotics or antidepressants. Related questions targeted our secondary objectives, assessing exercises, perceptions of obesity, and its impact on mental health status.

The second section incorporated two major tools to assess mental health status: PHQ-2 for depression disorder and GAD-2 for anxiety screening, both widely recognized for their validity and reliability. Participants with positive results (scored 3 or more) underwent further assessment using PHQ-9 and GAD-7. The questionnaire was translated into Arabic using a back-to-back method.

Inclusion criteria: All adult Saudi individuals aged 18 years and older visiting primary care centers in the three healthcare clusters in Riyadh, Saudi Arabia.

Exclusion criteria: Non-Saudi individuals, population younger than 18 years, disabled or unstable patients, or those known to have severe psychiatric problems.

Statistical Considerations Data were entered using Excel and analyzed using IBM Corp. Released 2015. IBM SPSS Statistics for Windows, Version 23.0. Armonk, NY: IBM Corp. Frequencies and percentages describe categorical variables (e.g., gender, etc.). The Chi-square test and Fisher's exact test compared categorical variables, with a p-value <0.05 considered statistically significant. Assuming a response distribution of 50%, a 95% confidence level, and a 5% margin of error, the calculated sample size is 385 using Raosoft [30].

## Results

A total of 480 adult Saudi participants were included in the current study. Most of them were males (61.5%) and in the age group of 18 to 40 years (77.3%). Regarding their educational level, 298 (62.1%) of the participants completed their bachelor’s degree, and 128 (26.7%) were completing their postgraduate studies. The majority of the participants had a BMI of 18.5 to 24.9 and 25 to 29.9 (31.9% for each). The characteristics of the participants are summarized in Table 1.

**Table 1:** Socio-demographic characteristics of the study participants (n=480)

Variable	Categories	Frequency	Percent
Gender	Male	295	61.5
	Female	185	38.5
Age (in years)	18 to 40	371	77.3
	More than 40	109	22.7
Educational level	Illiterate	2	0.4
	Intermediate	5	1
	Secondary	47	9.8
	Bachelor’s degree	298	62.1
	Postgraduate	128	26.7
BMI (kg/m^2^)	Less than 18.5	15	3.1
	18.5 to 24.9	153	31.9
	25 to 29.9	150	31.3
	30 to 34.9	72	15
	35 to 39.9	43	9
	40 or above	47	9.8

**Figure 1:**
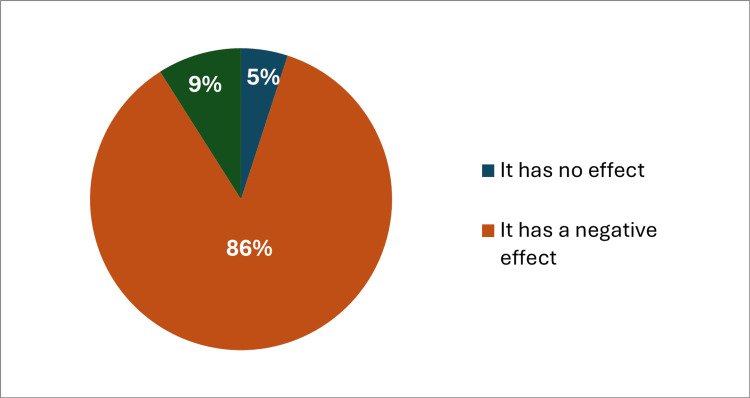
Do you think that obesity has a role and impact on quality of life and mental health?

**Figure 2:**
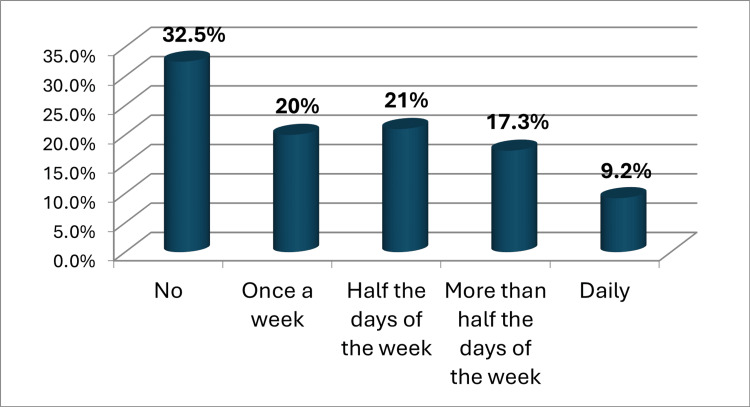
Is there time to devote to exercising regularly? (such as walking, swimming, jumping rope, etc.)

Regarding the history of psychiatric illness, 63 (13.1%) of the participants had a first-degree family history of psychiatric illness, and 51 (10.6%) had a previous medical history of psychiatric illness, while a lower percent (4%) used psychological treatments. 413 (86%) of the participants think that obesity has a negative effect on quality of life and mental health (Figure 1). The vast majority of the participants (94.6%) recommended having awareness campaigns and motivational seminars on a regular basis about obesity, its harms, and methods of treating it. Nearly a third of the participants (32.5%) had no time to devote to exercising regularly, and 101 (21%) exercised half the days of the week (Figure 2). The presence of sports clubs, walking areas, or parks near home enhances the practice of sports and the quality of life of 446 (92.9%) of the participants, and 471 (98.1%) of the participants think that losing weight and treating obesity will play a role in improving the quality of life and mental health in general (Table 2). 

**Table 2:** History of psychiatric illness and participants' perceptions about the relationship between obesity, quality of life, and mental health in general

Variable	Yes	No
	n (%)	
Is there any first-degree family history of psychiatric illness?	63 (13.1)	417 (86.9)
Have you previously been diagnosed with any psychiatric illness?	51 (10.6)	429 (89.4)
Are you using any psychological treatments?	19 (4)	461 (96)
Do you recommend having awareness campaigns and motivational seminars on a regular basis about obesity, its harms, and methods of treating it?	454 (94.6)	26 (5.4)
In your opinion, will the presence of sport clubs, walking areas, or parks near your home enhance your practice of sports and your quality of life?	446 (92.9)	34 (7.1)
Do you think that losing weight and treating obesity will play a role in improving the quality of life and mental health in general?	471 (98.1)	9 (1.9)

In regard to the prevalence of mental health issues, according to the PHQ-2 score, 151 (31.5%) of the participants were positive; out of them, 47 (31.1%) had mild depression, and 36 (23.8%) had moderate depression. On the other hand, 147 (30.5%) of the participants were positive for GAD-2; out of them, 41 (27.9%) had mild anxiety, and the same percent (27.9%) had moderate anxiety (Table 3). 

**Table 3:** Prevalence of anxiety and depression

Depression	Score	N (%)
Positive participants with PHQ-2	≥ 3	151 (31.5)
Severity using PHQ-9		
None to minimal	0 to 4	25 (16.6)
Mild	5 to 9	47 (31.1)
Moderate	10 to 14	36 (23.8)
Moderately severe	15 to 19	24 (15.9)
Severe	20 to 27	19 (12.6)
Anxiety	Score	N (%)
Positive participants with GAD-2	≥ 3	147 (30.5)
Severity using GAD-7		
No to low risk	0 to 4	34 (23.1)
Mild	5 to 9	41 (27.9)
Moderate	10 to 14	41 (27.9)
Severe	+ 15	31 (21.1)

The relationship between gender, educational level, and mental health status was assessed. There was a significant association between gender and general mental health status (P = 0.005); females tend to have a higher level of mental health issues compared to males (48.1% vs. 35.3%). A significant association was also found between depression and anxiety in relation to educational level (P = 0.032 and 0.003, respectively); participants with bachelor's degrees or above had a higher level of moderate to severe depression and anxiety compared to those with secondary school education or less. Other variables did not reach the statistical significance level (Table 4). ﻿

**Table 4:** Association between gender, educational level, and mental health status N.B. F: p-value calculated using Fisher's exact test, other p-values calculated using the Chi-square test. * Significant p-value < 0.05.

Variable	Gender		P-value	Educational level		P-value
	Male (n=295)	Female (n=185)		Secondary or less (n=54)	Bachelor or above (n=426)	
History of psychiatric illness						
Having family history	38 (12.9)	25 (13.5)	0.842	6 (11.1)	57 (13.4)	0.642
Previous medical history	32 (10.8)	19 (10.3)	0.842	9 (16.7)	42 (9.9)	0.126
Use of psychiatric medications	13 (4.4)	6 (3.2)	0.525	2 (3.7)	17 (4)	1.000^F^
Having mental health problem (+ve PHQ-2/GAD2)	104 (35.3)	89 (48.1)	0.005*	22 (40.7)	171 (40.1)	0.933
Current mental health status						
Depression (PHQ-9)						
None/mild	37 (46.8)	35 (48.6)	0.827	14 (70)	58 (44.3)	0.032*
Moderately/severe	42 (53.2)	37 (51.4)		6 (30)	73 (55.7)	
Anxiety (GAD-7)						
No/mild	35 (45.5)	40 (57.1)	0.157	15 (83.3)	60 (46.5)	0.003*
Moderate/severe	42 (54.5)	30 (42.9)		3 (16.7)	69 (53.5)	

We found that family history of psychiatric illness was significantly associated with previous medical history of psychiatric illness and use of psychiatric medications (P < 0.001). A previous medical history of psychiatric illness was significantly associated with the use of psychiatric medications (P < 0.001), and it was also associated with having a mental health problem (P = 0.024). Also, there was a significant association between depression, anxiety, and a previous history of psychiatric illness (P = 0.009 and 0.026, respectively); moderate/severe anxiety and depression were associated with having a previous history of psychiatric illness (Table 5).﻿

**Table 5:** Association between history of psychiatric illness and mental health status N.B. F: p value calculated using Fisher's exact test; other p-values calculated using the Chi-square test. * Significant p value < 0.05.

Variable	Family history of Psychiatric illness		P-value	Previous history of Psychiatric illness		P-value
	Yes (n=63)	No (n=417)		Yes (n=51)	No (n=429)	
History of psychiatric illness						
Having family history	-	-	-	23 (45.1)	40 (9.3)	< 0.001*
Previous medical history	23 (36.5)	28 (6.7)	< 0.001*	-	-	-
Use of psychiatric medications	9 (14.3)	10 (2.4)	< 0.001^F^*	16 (31.4)	3 (0.7)	< 0.001^F^*
Having mental health problem (+ve PHQ-2/GAD2)	28 (44.4)	165 (39.6)	0.462	28 (54.9)	165 (38.5)	0.024*
Current mental health status						
Depression (PHQ-9)						
None/mild	7 (30.4)	65 (50.8)	0.072	6 (24)	66 (52.4)	0.009*
Moderately/severe	16 (69.6)	63 (49.2)		19 (76)	60 (47.6)	
Anxiety (GAD-7)						
No/mild	12 (50)	63 (51.2)	0.913	6 (28.6)	69 (54.8)	0.026*
Moderate/severe	12 (50)	60 (48.8)		15 (71.4)	57 (45.2)	

When we assessed the relationship between physical activity and mental health status, the results revealed that having a previous history of psychiatric illness was significantly associated with no or once-a-week physical activity (P = 0.032). Also, there was a significant association between physical activity and depression (P < 0.001); moderate/severe depression was associated with no or once a week physical activity (Table 6). ﻿

**Table 6:** Association between physical activity and mental health status

Variable	Physical activity		P-value
	No/Once a week (n=252)	Half the days of week or more/Daily (n=228)	
History of psychiatric illness			
Previous medical history	34 (13.5)	17 (7.5)	0.032*
Use of psychiatric medications	12 (4.8)	7 (3.1)	0.342
Having mental health problem (+ve PHQ-2/GAD2)	110 (43.7)	83 (36.4)	0.106
Current mental health status			
Depression (PHQ-9)			
None/mild	30 (35.7)	42 (62.7)	0.001*
Moderate/severe	54 (64.3)	25 (37.3)	
Anxiety (GAD-7)			
No/mild	40 (47.1)	35 (56.5)	0.261
Moderate/severe	45 (52.9)	27 (43.5)	

The relationship between BMI and gender, physical activity, and mental health status was assessed. There was a significant association between gender and BMI (P < 0.001), with females having a higher BMI compared to males. There was also a significant association between BMI and medical history of psychiatric illness (P = 0.026); having a previous medical history of psychiatric illness was associated with a higher BMI of 25 to 29.9 and 35 to 39.9. Other variables did not reach the statistical significance level (Table 7). 

**Table 7:** Association between BMI and gender, physical activity, and mental health status N.B. F: p-value calculated using Fisher's exact test; other p-values calculated using the Chi-square test. * Significant p-value < 0.05.

Variable	BMI (kg/m^2^)						P-value
	< 18.5 (n=15)	18-24.9 (n=153)	25-29.9 (n=150)	30-34.9 (n=72)	35-39.9 (n=43)	≥ 40 (n=47)	
Gender							
Male	4 (26.7)	92 (60.1)	110 (73.3)	46 (63.9)	21 (48.8)	22 (46.8)	< 0.001*
Female	11 (73.3)	61 (39.9)	40 (26.7)	26 (36.1)	22 (51.2)	25 (53.2)	
Physical activity							
No	4 (26.7)	46 (30.1)	47 (31.3)	28 (38.9)	17 (39.5)	14 (29.8)	0.285
Once a week	2 (13.3)	28 (18.3)	31 (20.7)	15 (20.8)	8 (18.6)	12 (25.5)	
Half the days of the week	4 (26.7)	35 (22.9)	29 (19.3)	16 (22.2)	10 (23.3)	7 (14.9)	
More than half the days of the week	2 (13.3)	28 (18.3)	35 (23.3)	9 (12.5)	4 (9.3)	5 (10.6)	
Daily	3 (20)	16 (10.5)	8 (5.3)	4 (5.6)	4 (9.3)	9 (19.1)	
History of psychiatric illness							
Having family history	2 (13.3)	21 (13.7)	24 (16)	12 (16.7)	3 (7)	1 (2.1)	0.139
Previous medical history	0 (0)	10 (6.5)	22 (14.7)	7 (9.7)	9 (20.9)	3 (6.4)	0.026^F^*
Use of psychiatric medications	0 (0)	6 (3.9)	8 (5.3)	2 (2.8)	2 (4.7)	1 (2.1)	0.825^F^
Having mental health problem (+ve PHQ-2/GAD2)	8 (53.3)	62 (40.5)	56 (37.3)	28 (38.9)	20 (46.5)	19 (40.4)	0.797
Current mental health status							
Depression (PHQ-9)							
None/mild	2 (40)	25 (54.3)	16 (35.6)	10 (41.7)	9 (52.9)	10 (71.4)	0.200
Moderately/severe	3 (60)	21 (45.7)	29 (64.4)	14 (58.3)	8 (47.1)	4 (28.6)	
Anxiety (GAD-7)							
No/mild	3 (42.9)	25 (51)	23 (51.1)	6 (40)	7 (43.8)	11 (73.3)	0.515
Moderate/severe	4 (57.1)	24 (49)	22 (48.9)	9 (60)	9 (56.3)	4 (26.7)	

The relationship between BMI and mental health status in relation to gender was tested. Results showed that there was a significant association between BMI and previous medical history of psychiatric illness among females (P = 0.044); a BMI of 35 to 39.9 was associated with having a previous medical history of psychiatric illness. Other variables did not show any significance with BMI (Table 8). ﻿

**Table 8:** Association between BMI and mental health status is stratified by gender N.B. F: p-value calculated using Fisher's exact test; other p-values calculated using the Chi-square test. * Significant p-value < 0.05.

Variable	BMI (kg/m^2^)						P-value
	< 18.5 (n=15)	18.5-24.9 (n=153)	25-29.9 (n=150)	30-34.9 (n=72)	35-39.9 (n=43)	≥ 40 (n=47)	
	Males (n=295)						
History of Psychiatric illness							
Having family history	0 (0)	9 (9.8)	17 (15.5)	9 (19.6)	3 (14.3)	0 (0)	0.203^F^
Previous medical history	0 (0)	6 (6.5)	16 (14.5)	5 (10.9)	3 (14.3)	2 (9.1)	0.514^F^
Use of psychiatric medications	0 (0)	4 (4.3)	6 (5.5)	1 (2.2)	1 (4.8)	1 (4.5)	0.951^F^
Mental health status by screening tool:							
Positive PHQ-2 (≥ 3)	1 (25)	27 (29.3)	28 (25.5)	13 (28.3)	4 (19)	6 (27.3)	0.953
Positive GAD-2 (≥ 3)	1 (25)	30 (32.6)	31 (28.2)	6 (13)	5 (23.8)	4 (18.2)	0.213
Having mental health problem (+ve PHQ-2/GAD2)	2 (50)	38 (41.3)	37 (33.6)	14 (30.4)	7 (33.3)	6 (27.3)	0.685
Current mental health status							
Depression (PHQ-9)							
None/mild	1 (100)	13 (48.1)	10 (35.7)	6 (46.2)	3 (75)	4 (66.7)	0.463^F^
Moderately/severe	0 (0)	14 (51.9)	18 (64.3)	7 (53.8)	1 (25)	2 (33.3)	
Anxiety (GAD-7)							
No/mild	1 (100)	12 (40)	16 (51.6)	1 (16.7)	2 (40)	3 (75)	0.364^F^
Moderate/severe	0 (0)	18 (60)	15 (48.4)	5 (83.3)	3 (60)	1 (25)	
	Females (n=185)						
History of psychiatric illness							
Having family history	2 (18.2)	12 (19.7)	7 (17.5)	3 (11.5)	0 (0)	1 (4)	0.141^F^
Previous medical history	0 (0)	4 (6.6)	6 (15)	2 (7.7)	6 (27.3)	1 (4)	0.044^F^*
Use of psychiatric medications	0 (0)	2 (3.3)	2 (5)	1 (3.8)	1 (4.5)	0 (0)	0.948^F^
Mental health status by screening tool							
Positive PHQ-2 (≥ 3)	4 (36.4)	19 (31.1)	17 (42.5)	11 (42.3)	13 (59.1)	8 (32)	0.288
Positive GAD-2 (≥ 3)	6 (54.5)	19 (31.1)	14 (35)	9 (34.6)	11 (50)	11 (44)	0.479
Having mental health problem (+ve PHQ-2/GAD2)	6 (54.5)	24 (39.3)	19 (47.5)	14 (53.8)	13 (59.1)	13 (52)	0.605
Current mental health status							
Depression (PHQ-9)							
None/mild	1 (25)	12 (63.2)	6 (35.3)	4 (36.4)	6 (46.2)	6 (75)	0.260^F^
Moderately/severe	3 (75)	7 (36.8)	11 (64.7)	7 (63.6)	7 (53.8)	2 (25)	
Anxiety (GAD-7)							
No/mild	2 (33.3)	13 (68.4)	7 (50)	5 (55.6)	5 (45.5)	8 (72.7)	0.510^F^
Moderate/severe	4 (66.7)	6 (31.6)	7 (50)	4 (44.4)	6 (54.5)	3 (27.3)	

## Discussion

Studying the association and impact of obesity on health and psychological well-being in patients with obesity is crucial, especially its correlation to depression and anxiety, subsequently affecting the quality of life [12].

Regarding the demographic characteristics of the participants, the majority were males (61.5%). More than half (62.1%) of the participants had completed their bachelor’s degree. About two-thirds of the participants had a BMI higher than 25. Concerning the history of psychiatric illness, 13.1% of the participants had a first-degree family history of psychiatric illness, and 51 (10.6%) had a previous medical history of psychiatric illness. These findings were consistent with those reported in parallel studies, which showed a slightly higher prevalence [13-14]. The vast majority (86%) of the participants believe that obesity has a negative effect on quality of life and mental health. This sentiment aligns with congruent studies, one reporting the impact of obesity on quality of life and another conducted in Saudi Arabia, which reported the correlation between obesity and emotional, social, and behavioral problems associated with physical limitations, also mentioning the negative effects of obesity [15].

Concerning the prevalence of mental health issues, according to the PHQ-2 score, 31.5% of the participants tested positive. Out of them, 31.1% were experiencing mild depression, and the rest (23.8%) had moderate depression. This aligns with findings from an analogous study, which reported that 32% of obese people are more likely to have depression [15]. Similar results were mentioned in a study conducted in Saudi Arabia in the eastern province, where 41.7% of obese participants had depression [16]. One-third (30.5%) of the participants tested positive using the GAD-2, with 27.9% experiencing mild anxiety and the same percentage (27.9%) had moderate anxiety. This consistency was found in the results of a study conducted in Greece and another study in Saudi Arabia, which reported higher levels of anxiety in obese patients compared to other groups [17]. Additionally, prevalent psychological disorders, including depression, anxiety, and stress, were mentioned among obese participants in a Saudi Arabian study conducted in Abha, with the prevalence of depression in obese participants being 48.1%, anxiety at 58.9%, and stress found in 40.4% of the participants [18].

There was a significant association between gender and mental health status, with females tending to have a higher level of mental health issues compared to males. Analogous findings were mentioned in congruent studies [19-22]. Additionally, it was found that a family history of psychiatric illness was significantly associated with a previous medical history of psychiatric illness and the use of psychiatric medications. This was also reported in the parallel study conducted by Ali et al., where the association between a family history of psychiatric disorders and major psychiatric disorders was found [23].

A statistically significant association was found between physical activity and depression; moderate/severe depression was associated with no or once-a-week physical activity, and this was found to be in agreement with the findings of the studies [24-26].

Gender and BMI were found to be significantly associated, with females having a higher BMI compared to males. This observation is closely related to the findings reported in the study by Cooper et al. and other studies [27,28].

The results also revealed a significant association between BMI and the previous medical history of psychiatric illness among females, where a BMI of 35 to 39.9 was linked to having a prior medical history of psychiatric illness. Additionally, there was a significant association between BMI and the overall medical history of psychiatric illness; having a previous medical history of psychiatric issues was associated with higher BMI values in the ranges of 25 to 29.9 and 35 to 39.9. These findings are consistent with what was reported in the Simon study [29].

Limitations: This study has a limited sample size for specific BMI categories and relies on screening tools for mental health disorders, primarily depression and anxiety, rather than adopting holistic approaches. Achieving a comprehensive assessment would necessitate a larger research study.

## Conclusions

The study revealed a significant association between obesity and elevated BMI, with a higher prevalence of both depression and anxiety among the participants. Approximately one-third of the total participants exhibited symptoms of depression, while an additional one-third manifested symptoms of an anxiety disorder. Importantly, the study emphasized the significance of a family history of psychiatric disorders as a crucial factor contributing to the heightened prevalence of specific psychiatric conditions. These findings underscore the imperative need for targeted interventions addressing obesity and its potential impact on mental health, particularly when considering the context of familial psychiatric history.

Recommendations: Further research endeavors and heightened awareness initiatives aimed at developing effective strategies to alleviate the dual burden of obesity and mental health disorders, thereby fostering comprehensive well-being as a part of Vision 2030, encompassing initiatives such as exercises to lose weight, protective measures, new facilities, sports projects, and events that promote a healthy lifestyle, contributing to comprehensive well-being.
